# Parallel selection on ecologically relevant gene functions in the transcriptomes of highly diversifying salmonids

**DOI:** 10.1186/s12864-019-6361-2

**Published:** 2019-12-23

**Authors:** Kevin Schneider, Colin E. Adams, Kathryn R. Elmer

**Affiliations:** 1 Institute of Biodiversity, Animal Health & Comparative Medicine, College of Medical, Veterinary & Life Sciences, University of Glasgow, Glasgow, G12 8QQ UK; 2 Scottish Centre for Ecology and the Natural Environment, University of Glasgow, Rowardennan, G63 0AW UK

**Keywords:** Molecular evolution, Adaptation, Freshwater fishes, Diversification, Relaxed selection, Selective pressure, Purifying selection, Positive selection, Transcriptomics, Gene ontology

## Abstract

**Background:**

Salmonid fishes are characterised by a very high level of variation in trophic, ecological, physiological, and life history adaptations. Some salmonid taxa show exceptional potential for fast, within-lake diversification into morphologically and ecologically distinct variants, often in parallel; these are the lake-resident charr and whitefish (several species in the genera *Salvelinus* and *Coregonus*). To identify selection on genes and gene categories associated with such predictable diversifications, we analysed 2702 orthogroups (4.82 Mbp total; average 4.77 genes/orthogroup; average 1783 bp/orthogroup). We did so in two charr and two whitefish species and compared to five other salmonid lineages, which do not evolve in such ecologically predictable ways, and one non-salmonid outgroup.

**Results:**

All selection analyses are based on *Coregonus* and *Salvelinus* compared to non-diversifying taxa. We found more orthogroups were affected by relaxed selection than intensified selection. Of those, 122 were under significant relaxed selection, with trends of an overrepresentation of serine family amino acid metabolism and transcriptional regulation, and significant enrichment of behaviour-associated gene functions. Seventy-eight orthogroups were under significant intensified selection and were enriched for signalling process and transcriptional regulation gene ontology terms and actin filament and lipid metabolism gene sets. Ninety-two orthogroups were under diversifying/positive selection. These were enriched for signal transduction, transmembrane transport, and pyruvate metabolism gene ontology terms and often contained genes involved in transcriptional regulation and development. Several orthogroups showed signs of multiple types of selection. For example, orthogroups under relaxed and diversifying selection contained genes such as *ap1m2*, involved in immunity and development, and *slc6a8*, playing an important role in muscle and brain creatine uptake. Orthogroups under intensified and diversifying selection were also found, such as genes *syn3*, with a role in neural processes, and *ctsk*, involved in bone remodelling.

**Conclusions:**

Our approach pinpointed relevant genomic targets by distinguishing among different kinds of selection. We found that relaxed, intensified, and diversifying selection affect orthogroups and gene functions of ecological relevance in salmonids. Because they were found consistently and robustly across charr and whitefish and not other salmonid lineages, we propose these genes have a potential role in the replicated ecological diversifications.

## Background

Identifying the molecular mechanisms underlying adaptive phenotypic divergence is a central challenge for evolutionary biology; a key first step is to detect genes under selection rather than reflecting background neutral evolutionary processes. Parallel or convergent evolution at the molecular level may, or may not, be associated with phenotypic parallelisms across species, but the idea remains compelling [1–3] and has been an important analytical framework to advance research in non-model systems [4–6]. Molecular parallelism or convergence can be inferred either from nucleotide site-specific changes [5, 7–9] or at a higher level, in the sense of similar genes being targeted by similar selective forces [10–13].

Fishes have proven a powerful ecological and evolutionary group for comparisons of genes under selection and that are associated with ecological and evolutionary novelty. Sticklebacks, for example, have become a model group of repeated ecological adaptation of Holarctic marine and freshwater distribution [14–16]. In cichlid fishes, adaptive potential and highly malleable phenotypes are spread throughout the family. In some cases, it has been shown that relaxed selection [17–20] or positive selection [21, 22] correlate with phenotypic diversification. However, ecological opportunity differs dramatically among cichlid lineages [23–27], which makes it difficult to pinpoint taxa in which adaptive potential is elevated due to a shared genetic toolset [3, 6]. In contrast, freshwater lake-resident salmonids of different species and genera have similar ecological opportunity and commonly sympatric distributions across the northern hemisphere [28–30]. Furthermore, the freshwater habitats of northern fishes were all colonised on a similar postglacial timescale [30, 31], unlike the dramatically different and complex colonisation histories of cichlids [4, 32–34].

Salmonid fishes are increasingly used as model organisms in evolutionary research, because of their ecological diversity, economic value, and replicated evolution of distinct ecomorphs and traits in some taxa, such as depth specialisation and alternative migratory tactics [35–42]. Two salmonid genera in particular, the whitefishes (*Coregonus*) and the charrs (*Salvelinus*), are not sister taxa but exhibit parallel (or convergent) adaptive tendencies in freshwaters across the northern hemisphere. They have repeatedly diverged into various within-lake ecomorphs along the depth axis over short evolutionary time spans that are unmatched in any other salmonid species [2, 43–47]. The evolutionary and molecular basis for why *Coregonus* and *Salvelinus* show such a high degree of ecomorphological adaptability while other salmonid species do not is, however, unknown [39, 46, 47].

Determining how single and combined effects of selection act at the molecular level is facilitated by new analytical tools [48–50]. These selective processes are associated with adaptive evolution in different ways and are most powerful when linked with known lineage-specific phenotype changes or phenotypic diversification [51, 52]. Two selective processes – relaxed and intensified selection – are on opposite ends of the spectrum. Relaxed selection decreases the selective constraints of a gene and can lead to the accumulation of nonsynonymous substitutions and consequently changes in the amino acid sequence of a protein. By releasing a gene of selective constraints, relaxed selection can potentially foster phenotypic novelty, plasticity, and evolutionary innovation [4, 19, 49, 53]. In contrast, intensified selection increases selective constraints but can also manifest as positive intensified selection leading to more differences at some sites of a gene [54, 55]. Intensified selection implies changes to a gene have strong fitness consequences [49]. Additionally, lineage-specific episodic diversifying selection, or positive selection, will leave other signatures at the sequence level, such as more nonsynonymous changes than expected under neutrality at a subset of positions in a gene on some branches in the phylogenetic tree (i.e., branch-site model) [50, 56]. While relaxed and intensified selection are antithetical, in either case diversifying (positive) selection can simultaneously act at a proportion of sites in a gene [57, 58].

It has long been proposed that the propensity for ecological speciation in some salmonid lineages is associated with shared patterns of relaxed or diversifying selection on ecologically relevant genes and gene function terms [2, 3, 19, 39, 59, 60]. We focus on the well-characterised and richly diversifying genera *Coregonus* and *Salvelinus*, which show repeated within-lake divergences into distinct ecomorphs across the northern hemisphere [46, 47]. The *Coregonus* species assessed are lake resident at least since postglacial times and have high rates of within-lake adaptive divergence [45, 46, 61]. *Salvelinus* species are mostly freshwater residents and have undergone frequent adaptive divergence into ecomorphs along the depth axis, predominantly within lakes [38, 47, 62, 63]. Representatives of all other major salmonid lineages, *Oncorhynchus*, *Salmo*, and *Thymallus*, were also included in the dataset; these are generally riverine or anadromous genera that do not extensively diversify within lakes [64, 65]. By assessing consistency across two non-sister lineages, *Coregonus* and *Salvelinus*, our approach mitigates against false positives. The focus on orthogroups within and across species, rather than single genes, alleviates the problem of differing relaxation of selective constraint in duplicated compared to non-duplicated or rediploidised genes [66], which is particularly important in salmonids due to the whole-genome duplication (WGD) that their common ancestor experienced 80–103 Mya [67–69].

Here, we use a genome-wide comparative approach to test for shared evidence of selection at particular categories of genes, gene functions, and gene ontology terms in the two highly diversifying lineages, *Coregonus* and *Salvelinus*, relative to all other major salmonid lineages. We test a comprehensive suite of 2702 orthologous protein-coding gene sets (orthogroups) for signals of parallel relaxed, intensified, and diversifying/positive selection in *Coregonus* and *Salvelinus* (average of 4.77 genes per orthogroup; 4.82 Mbp in total; average of 1783 aligned bp per orthogroup). By distinguishing among different kinds of selection in replicate across two independent lineages, our approach can pinpoint the action of selective pressure more accurately. We find that different types of selection target different gene sets and functions in salmonids, with novel and established ecological relevance for repeated, parallel diversification potential.

## Results

### Selection parameter distribution and number of orthogroups under selection

**Fig. 1:**
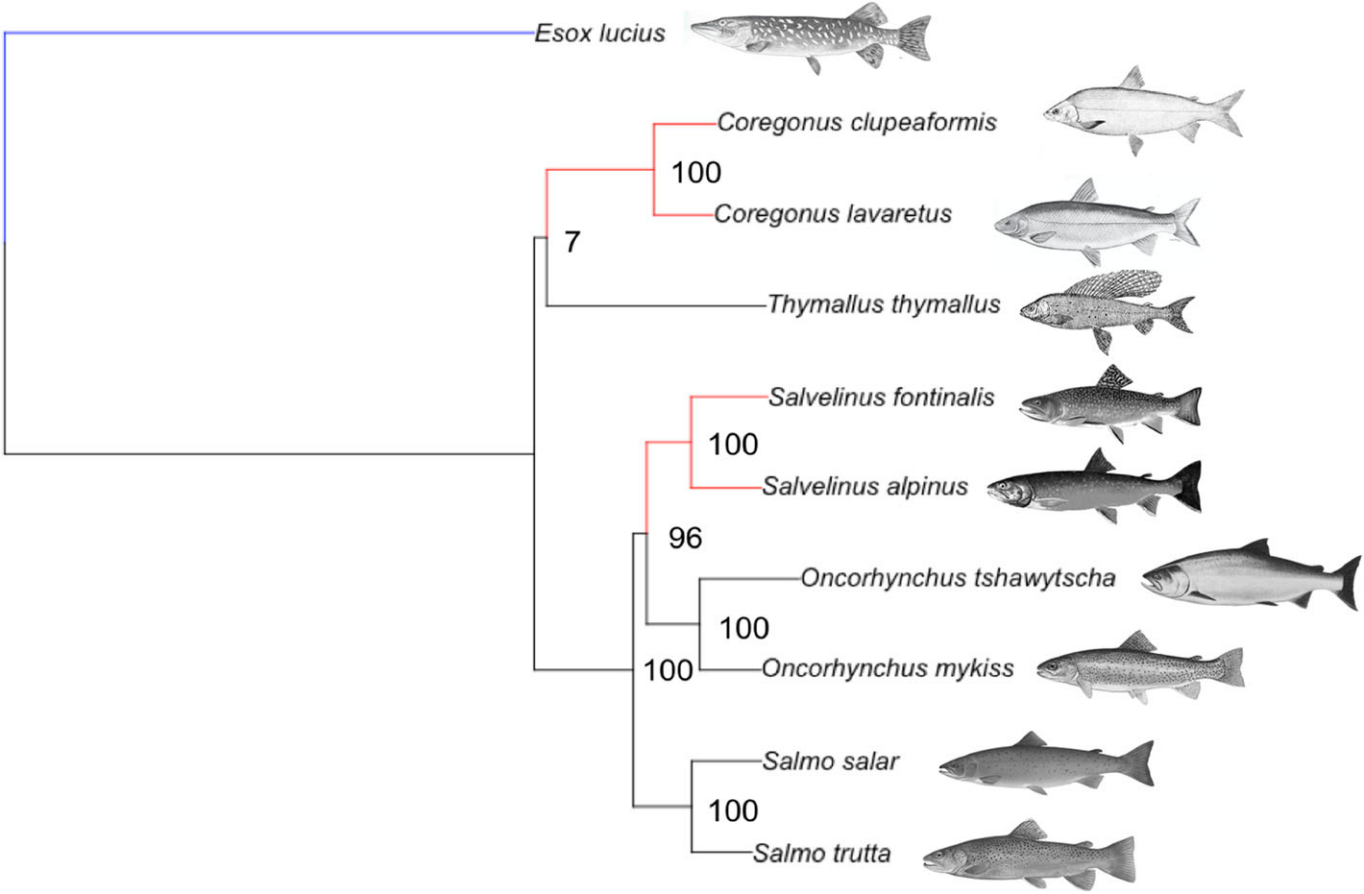
Maximum-likelihood phylogenetic tree of nine salmonid species (foreground taxa *Coregonus* and *Salvelinus* in red) and outgroup northern pike (*Esox lucius;* in blue). Node support values are bootstrap values from 1000 bootstrap replications. Branch lengths correspond to the number of substitutions per site. All pictures used here are under public domain. *Coregonus* and *Salvelinus* are two genera with exceptional ability for repeated, rapid diversification into ecomorphs within lakes that is unmatched in other salmonid taxa [2, 43–47]

**Fig. 2:**
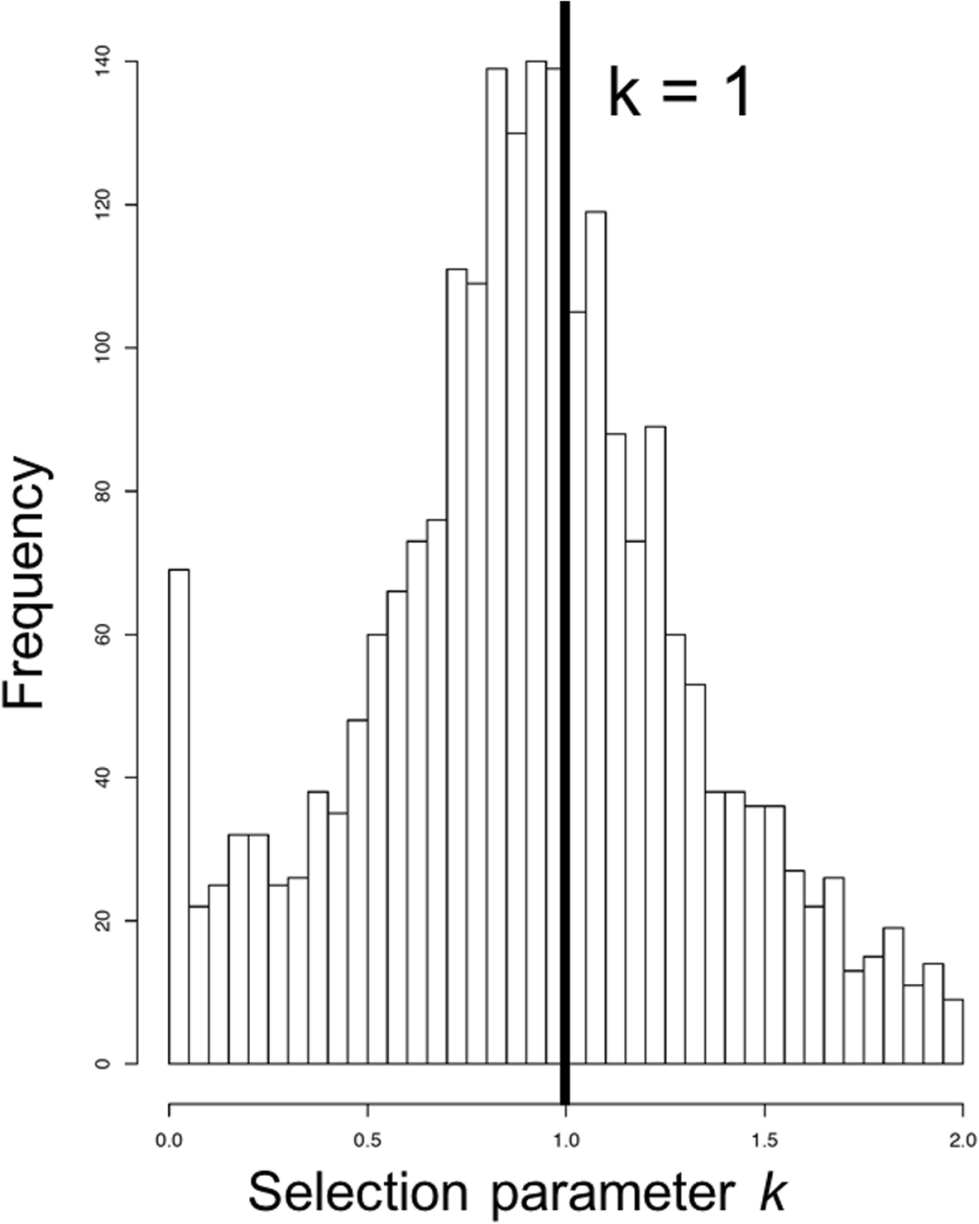
Histogram of the distribution of selection parameter *k* values (exponent of dN/dS ratio, i.e., *k* in ω^*k*^) from RELAX analysis in the 2702 orthogroups. The values shown are for *Coregonus* and *Salvelinus* compared to the other five salmonid species and the outgroup (pike). The neutral expectation of *k* = 1 is shown as a vertical line. *k* values above 2 are omitted for visibility purposes in the above plot (416 orthogroups have *k* values above 2; the maximum possible *k* value is 50; see Additional file 1 for total set of *k* values)

Shared molecular response to selection in two whitefish and two charr species was inferred relative to six background species (five salmonids and one pike, Fig. 1). The selection parameter *k* in whitefish and charr, ranging from 0 (very relaxed) to 50 (very intensified), had a median value of 0.992 across orthogroups and was significantly different from the neutral expectation of 1 (Wilcoxon signed-rank test: *V* = 1,995,900, *p* = 8.859E-06). Visually, there was an excess of orthogroups with *k* close to 0, indicating a high number of orthogroups under pronounced relaxed selection (Fig. 2). The number of orthogroups with *k* < 1 (1387; relaxed selection prevailing) was slightly higher than the number of orthogroups with *k* > 1 (1308; intensified selection prevailing), but not significantly so (Fisher’s Exact Test, *p* = 0.288).

On 2702 orthogroups in the final dataset we conducted analyses of relaxed and intensified selection (in *RELAX*) and diversifying/positive selection (in *aBSREL* with branch-site model). We inferred 138 orthogroups to be under relaxed selection (*k* < 1, false discovery rate (FDR) < 0.10) in either *Coregonus* or *Salvelinus*, of which 122 were found in both *Coregonus* and *Salvelinus*. On the other hand, 105 orthogroups showed signals of intensified selection (*k* > 1, FDR < 0.10), of which 78 included both *Coregonus* and *Salvelinus*. The number of relaxed orthogroups in *Coregonus* and *Salvelinus* was significantly higher compared to the number of intensified orthogroups (one-sided Fisher’s Exact Test, *p* = 0.035). Of the 2702 orthogroups, branch-site selection analyses inferred 111 orthogroups as being under significant diversifying/positive selection (FDR < 0.10), of which 92 included both *Coregonus* and *Salvelinus*. Thus, these orthogroups harbour a proportion of sites with significantly elevated dN/dS (= ω) values in at least one of the foreground branches leading to *Coregonus* or *Salvelinus* taxa.

**Fig. 3:**
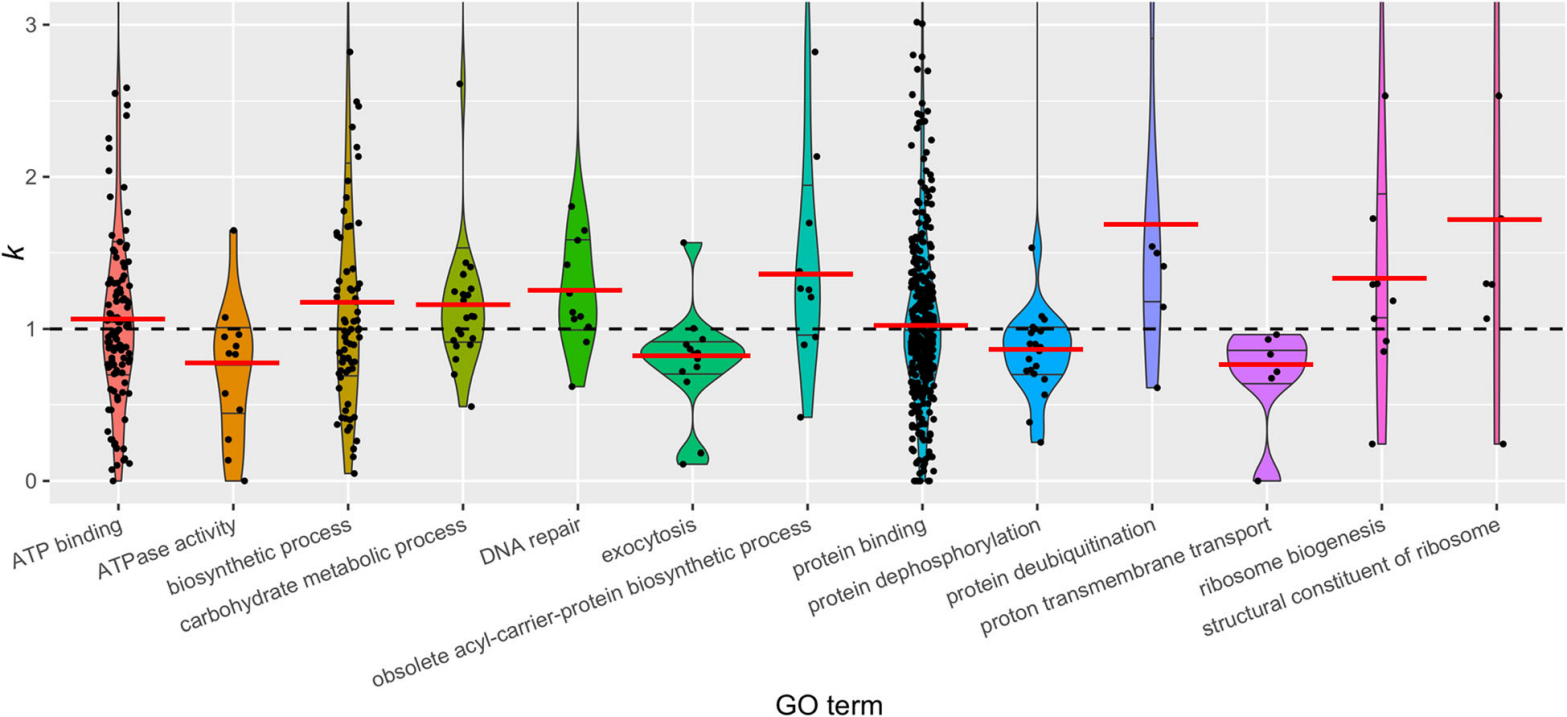
Selection parameter *k* distributions for the gene ontology (GO) terms that deviated from the null expectation in the total set of 2702 orthogroups. *k* values are selection parameter values (exponent of dN/dS ratio, i.e., *k* in ω^*k*^) in *Coregonus* and *Salvelinus* compared to the five other salmonid species and the outgroup (pike). The red horizontal bars indicate the medians of *k*

After averaging selection parameter values for each gene ontology (GO) term, 13 of 1478 GO terms showed significant deviations from the null expectation of *k* = 1 (Wilcoxon signed-rank tests: *p* < 0.05; Fig. 3). Eight of these had significantly elevated *k* values, indicating intensified selection. The other five had significantly lowered *k* values, which is evidence for relaxed selection. The GO term enrichment results agreed with the general shift of selection (distribution of *k*) in all orthogroups. The GO terms ‘carbohydrate metabolic process’ and ‘obsolete acyl-carrier-protein biosynthetic process’, for example, were also present in the orthogroups under intensified selection. The ‘ATPase activity’ and ‘proton transmembrane transport’ GO terms were also found among orthogroups under relaxed selection. Other deviating GO terms were ‘DNA repair’ and ‘protein deubiquitination’, with evidence for intensified selection, and ‘exocytosis’ and ‘protein dephosphorylation’, with evidence for relaxed selection (Fig. 3).

### Gene functions under relaxed selection

*Blast2GO* annotation and UniProt/Swiss-Prot literature research on the orthogroups under relaxed selection identified gene functions with potential relevance for the diversification process in charr and whitefish. Such functions included visual perception (e.g., ‘peripherin-2-like’), gene and gene product regulation (e.g., ‘E3 ubiquitin-protein ligase RNF128-like’), lipid metabolism (e.g., ‘calcium-independent phospholipase A2-gamma-like’), muscle and heart growth (e.g., ‘dual specificity protein phosphatase 6’), locomotion (e.g., ‘serine/threonine-protein phosphatase PP1-beta catalytic subunit’), and immunity (e.g., ‘adaptor-related protein complex 1’, ‘natterin-3-like’), but also genes with a role for various nervous system processes (e.g., ‘POU domain, class 4, transcription factor 3-like’; results of relaxed and intensified selection analyses: Additional file 1).

**Fig. 4:**
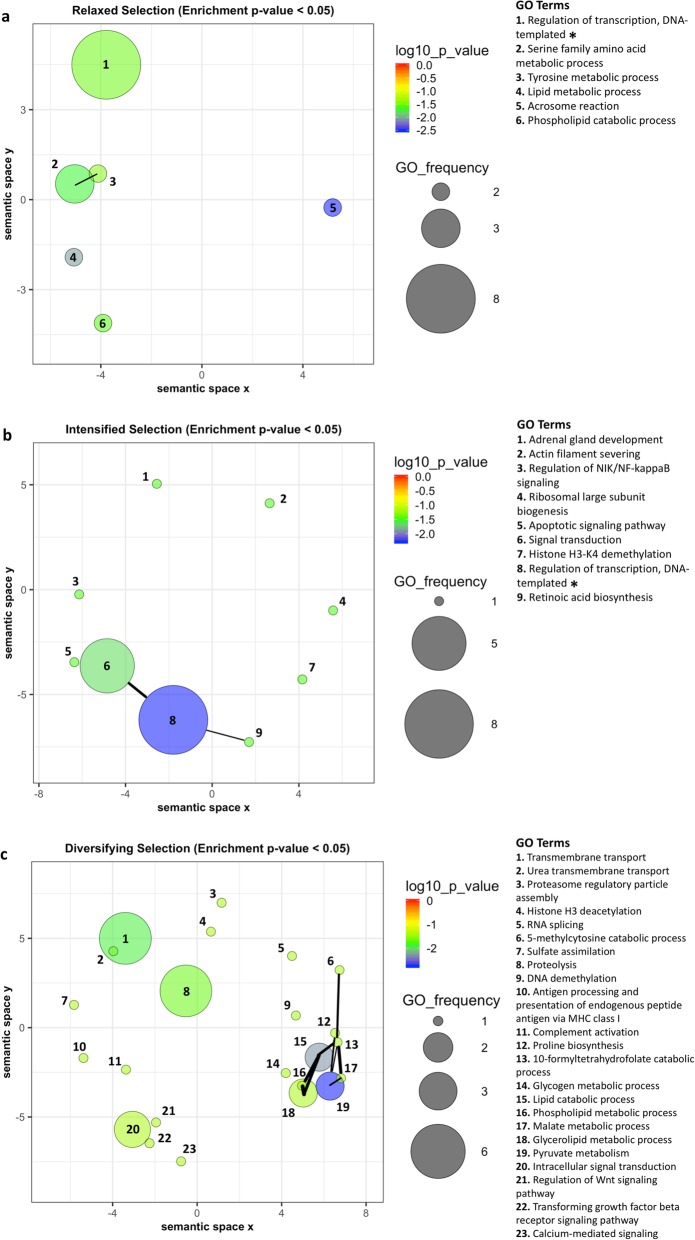
Gene Ontology (GO) terms with trends of overrepresentation (*p* < 0.05) in **a** the orthogroups under relaxed selection (FDR < 0.10), **b** the orthogroups under intensified selection (FDR < 0.10), and **c** the orthogroups under diversifying selection (FDR < 0.10). The orthogroups used are present in both *Coregonus* and *Salvelinus*. Bubble colour in the indicated colour spectrum corresponds to the log_10_ *p*-value of overrepresentation (blue = higher significance, red = lower significance). Bubble size corresponds to the frequency of a GO term in the orthogroups under selection. Highly similar GO terms are linked by edges in the graph, where the line width indicates the degree of similarity. The bubble placement corresponds to the position along two semantic space axes based on SimRel clustering as described in Material & methods [70]. Asterisks indicate overlap with enriched GO terms in orthogroups under other types of selection

We observed compelling trends of GO term enrichment (one-tailed Fisher’s Exact Tests, uncorrected *p* < 0.05 but FDR > 0.10) in the orthogroups under relaxed, intensified, and diversifying selection that largely agree with the research literature on the genes contained in those orthogroups (Fig. 4, Table 1). We found the 122 orthogroups under significant relaxed selection in *Coregonus* and *Salvelinus* were enriched for a total of 11 GO terms associated with transcriptional regulation, serine family amino acid metabolism, lipid metabolism, and oxidoreductase activity, amongst others (Table 1). The REVIGO redundancy analysis results showed transcriptional regulation, serine family amino acid metabolism, lipid metabolism, and acrosome reaction to be among the few non-redundant GO terms (frequency and significance plot of non-redundant GO terms: Fig. 4a, includes clustering by semantic similarity). Transcriptional regulation and serine family amino acid metabolism were the most frequent non-redundant GO terms. In total, six of 11 GO terms were found to be non-redundant.

**Table 1:** Over- and underrepresented terms from a GO enrichment analysis. Cor = *Coregonus*, Salv = *Salvelinus*, OGs = orthogroups

GO category	*p*-value	*p*-value (Cor + Salv)	# in OGs under selection	# in reference OGs	# in OGs under selection (Cor + Salv)	# in reference OGs (Cor + Salv)
Overrepresented GO terms – relaxed selection						
PTW/PP1 phosphatase complex	2.55E-03	3.03E-03	2	0	2	0
acrosome reaction	2.55E-03	3.03E-03	2	0	2	0
integral component of membrane	3.96E-03	1.77E-02	21	205	17	163
transcription factor complex	4.59E-03	5.52E-02	11	78	8	68
regulation of transcription, DNA-templated	6.29E-03	3.12E-02	10	70	8	60
DNA-binding transcription factor activity	1.22E-02	5.98E-02	9	66	7	57
regulation of transcription by RNA polymerase II	1.35E-02	2.20E-01	4	15	2	14
lipid metabolic process	1.43E-02	8.76E-03	2	2	2	1
protein serine/threonine kinase activity	2.00E-02	1.48E-02	3	9	3	7
protein tyrosine phosphatase activity	2.30E-02	2.71E-02	2	3	2	3
phospholipid catabolic process	2.30E-02	2.71E-02	2	3	2	3
tyrosine metabolic process	3.34E-02	3.92E-02	2	4	2	4
oxidoreductase activity, acting on paired donors, with incorporation or reduction of molecular oxygen	3.34E-02	2.71E-02	2	4	2	3
serine family amino acid metabolic process	3.70E-02	1.96E-02	3	12	3	8
nucleus	4.42E-02	6.75E-02	10	98	9	84
Overrepresented GO terms – intensified selection						
protein deubiquitination	1.73E-03	1.33E-01	3	4	1	3
regulation of transcription, DNA-templated	2.17E-03	5.18E-03	10	81	8	70
thiol-dependent ubiquitinyl hydrolase activity	8.36E-03	1.02E-01	2	2	1	2
DNA binding	2.48E-02	1.68E-02	8	85	7	70
steroid hormone receptor activity	2.71E-02	2.29E-02	2	5	2	5
acetyl-CoA carboxylase activity	3.85E-02	3.52E-02	1	0	1	0
acetyl-CoA carboxylase complex	3.85E-02	3.52E-02	1	0	1	0
Lys48-specific deubiquitinase activity	3.85E-02	1.00E+ 00	1	0	0	0
Sin3 complex	3.85E-02	3.52E-02	1	0	1	0
histone H3-K4 demethylation	3.85E-02	3.52E-02	1	0	1	0
retinoic acid biosynthetic process	3.85E-02	3.52E-02	1	0	1	0
NADP-retinol dehydrogenase activity	3.85E-02	3.52E-02	1	0	1	0
catalase activity	3.85E-02	1.00E+ 00	1	0	0	0
obsolete peroxidase reaction	3.85E-02	1.00E+ 00	1	0	0	0
response to oxidative stress	3.85E-02	1.00E+ 00	1	0	0	0
methane metabolic process	3.85E-02	1.00E+ 00	1	0	0	0
hydrogen peroxide catabolic process	3.85E-02	1.00E+ 00	1	0	0	0
palmitoyltransferase activity	3.85E-02	3.52E-02	1	0	1	0
beta1-adrenergic receptor activity	3.85E-02	1.00E+ 00	1	0	0	0
adenylate cyclase-activating G protein-coupled receptor signaling pathway	3.85E-02	1.00E+ 00	1	0	0	0
positive regulation of heart contraction	3.85E-02	1.00E+ 00	1	0	0	0
adrenergic receptor signaling pathway	3.85E-02	1.00E+ 00	1	0	0	0
adrenal gland development	3.85E-02	3.52E-02	1	0	1	0
corticotropin-releasing hormone binding	3.85E-02	1.00E+ 00	1	0	0	0
apoptotic signaling pathway	3.85E-02	3.52E-02	1	0	1	0
regulation of NIK/NF-kappaB signaling	3.85E-02	3.52E-02	1	0	1	0
iron-sulfur cluster binding	3.85E-02	3.52E-02	1	0	1	0
actin filament severing	3.85E-02	3.52E-02	1	0	1	0
dystrophin-associated glycoprotein complex	3.85E-02	3.52E-02	1	0	1	0
hexosaminidase activity	3.85E-02	1.00E+ 00	1	0	0	0
hyaluronic acid binding	3.85E-02	3.52E-02	1	0	1	0
ribosomal large subunit biogenesis	3.85E-02	3.52E-02	1	0	1	0
Overrepresented GO terms – diversifying selection						
transmembrane transporter activity	2.28E-02	1.19E-02	4	23	4	18
transmembrane transport	2.17E-02	1.25E-02	6	48	6	41
phospholipid metabolic process	4.11E-02	4.20E-02	1	0	1	0
regulation of Wnt signaling pathway	4.11E-02	4.20E-02	1	0	1	0
lipid phosphatase activity	4.11E-02	4.20E-02	1	0	1	0
proteolysis	3.20E-02	2.18E-02	6	53	6	47
acetyl-CoA carboxylase activity	4.11E-02	4.20E-02	1	0	1	0
pyruvate metabolic process	1.67E-03	1.75E-03	2	0	2	0
acetyl-CoA carboxylase complex	4.11E-02	4.20E-02	1	0	1	0
NAD-dependent histone deacetylase activity (H3-K14 specific)	4.11E-02	4.20E-02	1	0	1	0
histone H3 deacetylation	4.11E-02	4.20E-02	1	0	1	0
intracellular signal transduction	1.51E-02	4.27E-02	4	20	3	16
transforming growth factor beta receptor signaling pathway	4.11E-02	4.20E-02	1	0	1	0
serine-type endopeptidase activity	5.27E-03	4.56E-03	4	14	4	13
cytoskeleton	4.98E-02	4.14E-02	2	7	2	6
septin complex	4.11E-02	4.20E-02	1	0	1	0
complement activation	4.11E-02	4.20E-02	1	0	1	0
nociceptin receptor activity	4.11E-02	1.00E+ 00	1	0	0	0
calcium-mediated signaling	4.11E-02	4.20E-02	1	0	1	0
glycerolipid metabolic process	3.98E-02	4.14E-02	2	6	2	6
kinesin complex	4.11E-02	4.20E-02	1	0	1	0
kinesin binding	4.11E-02	4.20E-02	1	0	1	0
lipid catabolic process	4.88E-03	5.10E-03	2	1	2	1
oxidoreductase activity, acting on paired donors, with incorporation or reduction of molecular oxygen	2.25E-02	1.93E-01	2	4	1	4
L-ascorbic acid binding	1.54E-02	1.58E-01	2	3	1	3
lipoprotein lipase activity	1.67E-03	1.75E-03	2	0	2	0
Lys48-specific deubiquitinase activity	4.11E-02	1.00E+ 00	1	0	0	0
synapse	4.11E-02	4.20E-02	1	0	1	0
Sin3 complex	4.11E-02	4.20E-02	1	0	1	0
sulfate assimilation	4.11E-02	4.20E-02	1	0	1	0
adenylylsulfate kinase activity	4.11E-02	4.20E-02	1	0	1	0
sulfate adenylyltransferase (ATP) activity	4.11E-02	4.20E-02	1	0	1	0
glycogen metabolic process	4.11E-02	4.20E-02	1	0	1	0
antigen processing and presentation of endogenous peptide antigen via MHC class I	4.11E-02	4.20E-02	1	0	1	0
melatonin receptor activity	4.11E-02	1.00E+ 00	1	0	0	0
3-hydroxyisobutyrate dehydrogenase activity	4.11E-02	4.20E-02	1	0	1	0
NAD binding	1.54E-02	1.61E-02	2	3	2	3
urea transmembrane transporter activity	4.11E-02	4.20E-02	1	0	1	0
urea transmembrane transport	4.11E-02	4.20E-02	1	0	1	0
5-methylcytosine catabolic process	4.11E-02	4.20E-02	1	0	1	0
methylcytosine dioxygenase activity	4.11E-02	4.20E-02	1	0	1	0
DNA demethylation	4.11E-02	4.20E-02	1	0	1	0
pyrroline-5-carboxylate reductase activity	4.11E-02	4.20E-02	1	0	1	0
proline biosynthetic process	4.11E-02	4.20E-02	1	0	1	0
malate dehydrogenase (decarboxylating) (NAD+) activity	4.11E-02	4.20E-02	1	0	1	0
malate metabolic process	4.11E-02	4.20E-02	1	0	1	0
proteasome regulatory particle assembly	4.11E-02	4.20E-02	1	0	1	0
beta1-adrenergic receptor activity	4.11E-02	1.00E+ 00	1	0	0	0
adenylate cyclase-activating G protein-coupled receptor signaling pathway	4.11E-02	1.00E+ 00	1	0	0	0
positive regulation of heart contraction	4.11E-02	1.00E+ 00	1	0	0	0
adrenergic receptor signaling pathway	4.11E-02	1.00E+ 00	1	0	0	0
small ribosomal subunit	4.11E-02	4.20E-02	1	0	1	0
10-formyltetrahydrofolate catabolic process	4.11E-02	4.20E-02	1	0	1	0
formyltetrahydrofolate dehydrogenase activity	4.11E-02	4.20E-02	1	0	1	0
hydroxymethyl-, formyl- and related transferase activity	4.11E-02	4.20E-02	1	0	1	0
regulation of neuroinflammatory response	4.11E-02	1.00E+ 00	1	0	0	0
hexosaminidase activity	4.11E-02	1.00E+ 00	1	0	0	0
junctional membrane complex	4.11E-02	4.20E-02	1	0	1	0
Underrepresented GO terms – diversifying selection						
DNA binding	1.89E-02	3.46E-02	0	93	0	77

**Fig. 5:**
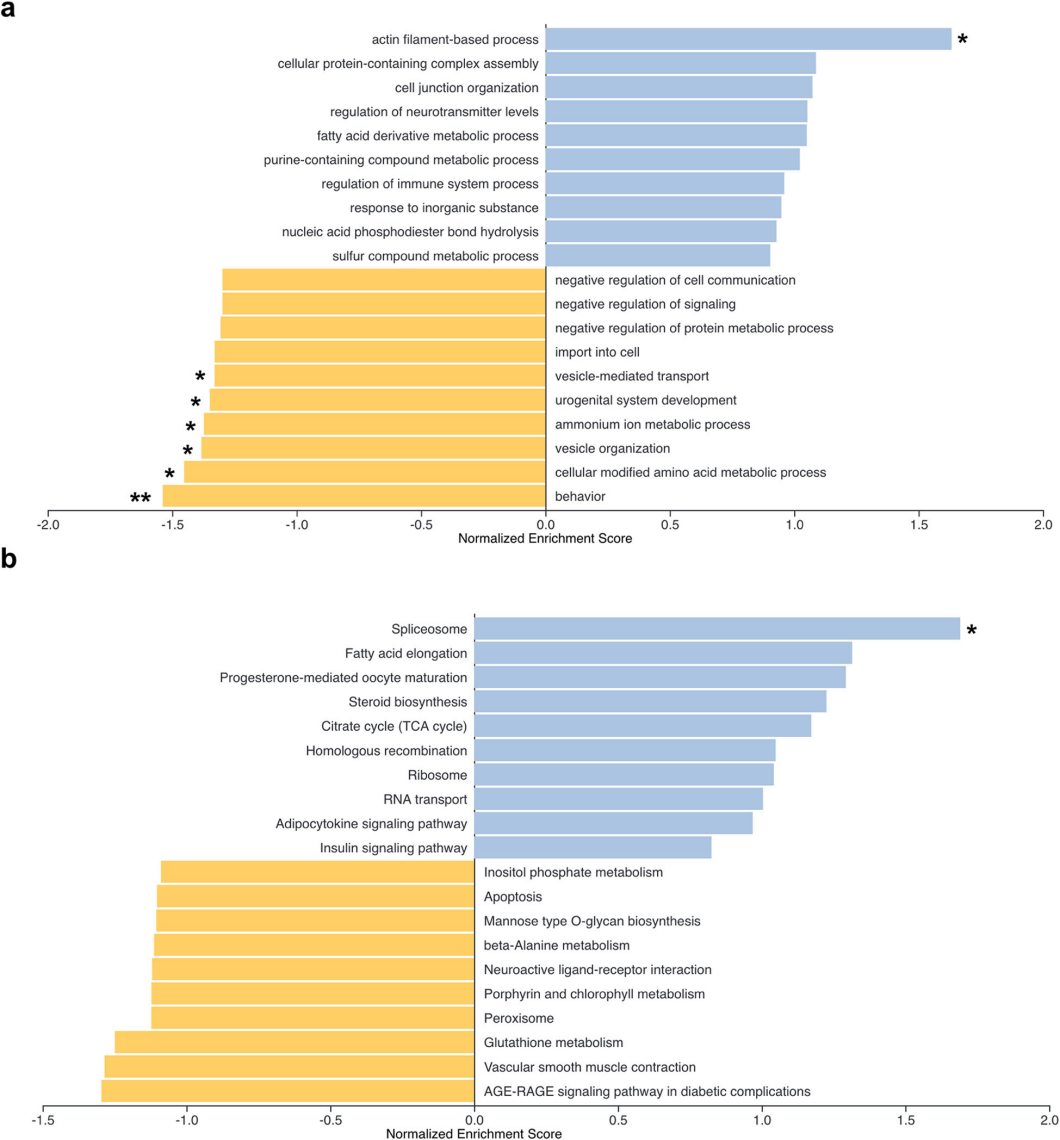
Top ten enriched **a** biological process GO terms and **b** KEGG pathways based on a gene set enrichment analysis (GSEA) of all orthogroups in *Coregonus* and *Salvelinus* relative to non-diversifying taxa. Terms with negative scores (yellow) were inferred to be under relaxed selection and with positive scores (blue) under intensified selection. The normalised enrichment score is calculated from transformed *k* values of each gene in a gene set. ** indicates significant gene set enrichment after multiple correction (FDR < 0.2); * indicates significant gene set enrichment without multiple correction ( 0.05)

Among the top ten enriched functions in relaxed orthogroups in both *Coregonus* and *Salvelinus*, behaviour and many neural function GO terms and KEGG pathways were found in gene set enrichment analyses (Fig. 5). This is in agreement with the neural process orthogroups and serine family amino acid metabolism GO terms obtained in the GO term enrichment analysis above. The behaviour gene set was the only gene set that was significantly enriched after FDR correction (Fig. 5). Other overrepresented functions included, for example, negative regulation of signalling, urogenital system development, the peroxisome pathway, vascular smooth muscle contraction, and the AGE-RAGE signalling pathway, which plays a major role in inflammation and infection.

### Gene functions under intensified selection

The gene functions of the 78 orthogroups under intensified selection in both *Coregonus* and *Salvelinus* (results of relaxed and intensified selection analyses: Additional file 1) were found from literature search to be frequently involved in functions relevant for lipid and carbohydrate metabolism (e.g., ‘acetyl-CoA carboxylase beta’ and ‘endoplasmic reticulum mannosyl-oligosaccharide 1,2-alpha-mannosidase-like’, respectively) as well as neurological and bone development (e.g., neurological development: ‘synapsin-3’, bone development: ‘cathepsin K precursor’ and ‘paired like homeodomain 1’).

The orthogroups under intensified selection in *Coregonus* and *Salvelinus* were enriched for transcriptional regulation GO terms, but also for those associated with ubiquitine-related processes and steroid hormone receptor activity, amongst others. A total of 18 GO terms were overrepresented (Table 1). Transcriptional regulation and several signalling processes were the only high-frequency GO terms among the few non-redundant GO terms in the *REVIGO* analysis (frequency and significance plot of non-redundant GO terms: Fig. 4b, includes clustering by semantic similarity). In total, nine of 18 GO terms remained after the *REVIGO* redundancy analysis.

In the gene set enrichment analysis of all intensified selection orthogroups present in *Coregonus* and *Salvelinus*, the ‘actin filament-based process’ GO term, the ‘spliceosome’ and several signalling KEGG pathways were among the top enriched functions (Fig. 5). Other functions included ‘cellular protein-containing complex assembly’, ‘fatty acid elongation’, ‘progesterone-mediated oocyte maturation’, and ‘steroid biosynthesis’. Overall, the gene enrichment results (Fig. 5) mostly agree with the GO term overrepresentations (Fig. 4b).

### Gene functions under diversifying selection

A large number of the 92 orthogroups under diversifying selection in *Coregonus* and *Salvelinus* were found in literature search to contain genes involved in regulation of gene expression, signal transduction and transmembrane transporter genes, but also immunity-related genes and a gene of the FOX set of genes, ‘FOX I1-ema’, a tissue-specific splicing factor important in otic placode formation and jaw development in zebrafish [71] (orthogroups under diversifying selection: Additional file 2, includes associated GO terms).

Orthogroups under diversifying selection were enriched for GO terms associated with transmembrane transport, phospholipid metabolic processes, acetyl-CoA carboxylase activity, various lipid metabolic processes, regulation of Wnt signalling pathway, and RNA splicing, amongst others (Fig. 4c, Table 1). A total of 47 GO terms were overrepresented, of which 23 remained after the *REVIGO* redundancy analysis. Pyruvate metabolism, several signal transduction processes, lipid metabolism, and transmembrane transport processes were shown to be amongst the non-redundant GO terms in the *REVIGO* analysis (frequency and significance plot of non-redundant GO terms: Fig. 4c, includes clustering by semantic similarity). Compared to the orthogroups under relaxed or intensified selection (Fig. 4a,b), the orthogroups under diversifying selection included a higher number of rather dissimilar low-frequency GO terms, apart from a cluster of similar metabolic GO terms (Fig. 4c). Only one GO term, ‘DNA binding’, was underrepresented (*p* < 0.05); with zero occurrences in the orthogroups under diversifying selection in *Coregonus* and *Salvelinus* but 77 occurrences in all other orthogroups.

### Overlap between selection types

**Fig. 6:**
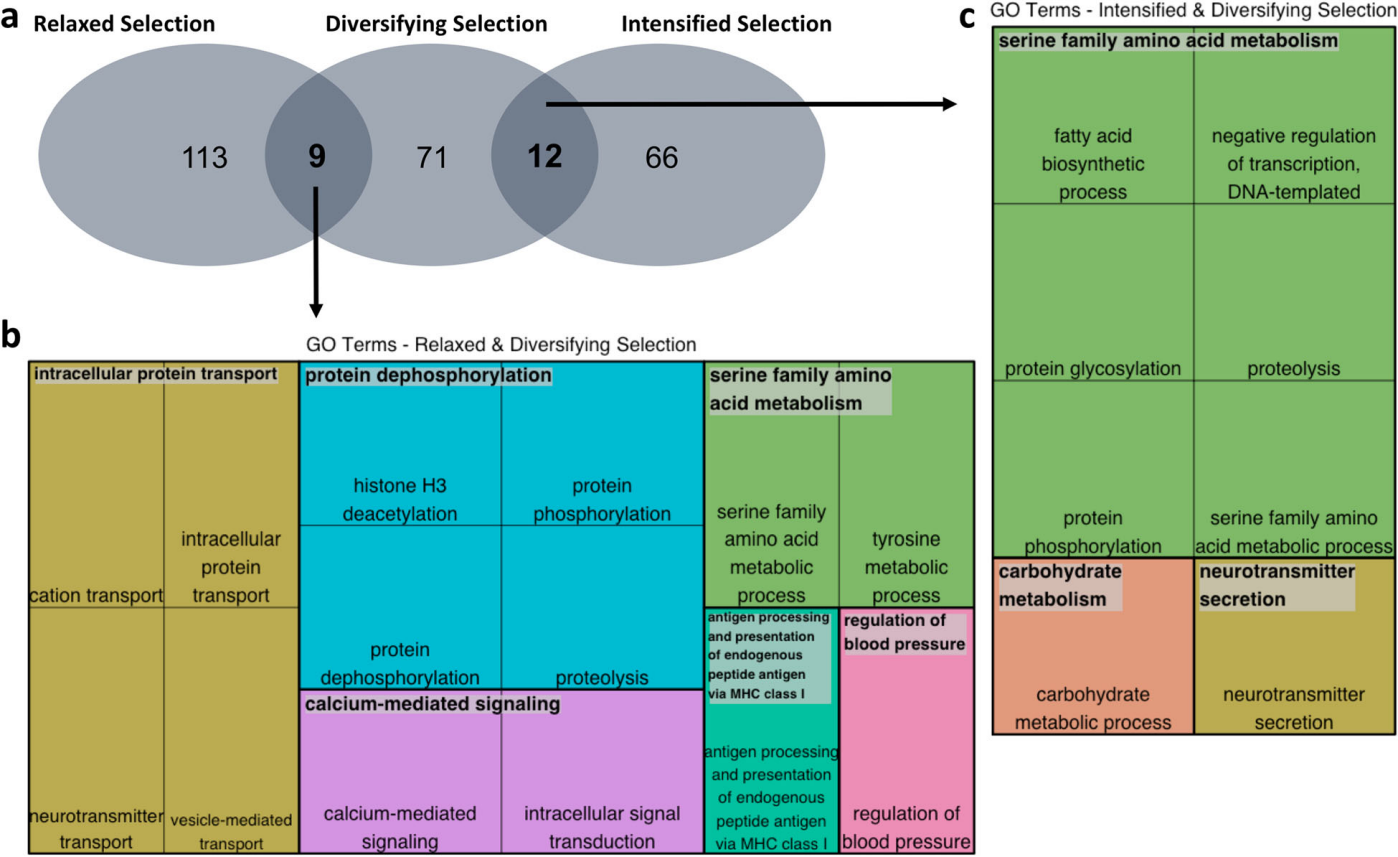
Overlapping orthogroups under selection and biological process GO terms. **a** Overlap between orthogroups under relaxed and diversifying or intensified and diversifying selection in both *Coregonus* and *Salvelinus*. **b** Biological process GO terms of orthogroups under both relaxed and diversifying selection. **c** Biological process GO terms of orthogroups under both intensified and diversifying selection. In **b** and **c**, rectangles are clustered into larger, coloured rectangles based on relationships among GO terms [70]

We identified nine orthogroups that showed both signals of relaxed selection (*RELAX*) and diversifying selection (*aBSREL*) and 12 orthogroups that showed both signals of intensified selection (*RELAX*) and diversifying selection (*aBSREL*) (Fig. 6a, Table 2). The overlap between orthogroups under relaxed and diversifying selection was higher than expected by chance, but not significantly so (hypergeometric expectation: 3.6 vs observed 9; one-tailed Fisher’s Exact Test: *p* = 0.126). The overlap between orthogroups under intensified and diversifying selection was significantly higher than expected by chance (hypergeometric expectation: 2.1 vs observed 12; one-tailed Fisher’s Exact Test: *p* = 0.004).

**Table 2:** Orthogroups (OGs) with signals of relaxed and diversifying selection or intensified and diversifying selection. *k* values are selection parameter values (exponent of dN/dS ratio, i.e., *k* in ω^*k*^). FDR values are from Benjamini-Hochberg FDR correction. Orthogroups shown are present in both *Coregonus* and *Salvelinus*. For aBSREL, *p*-values after correction for multiple testing (FDR < 0.10) are shown

OG ID	Gene description	GO terms (obtained with Blast2GO)	Additional information (manually derived from UniProt/Swiss-Prot)	FDR (RELAX)	*p* (aBSREL)	*k*
Relaxed & diversifying selection						
OG0001544	adaptor-related protein complex 1, mu 2 subunit (ap1m2)	intracellular protein transport; vesicle-mediated transport; clathrin adaptor complex	antigen processing and presentation of exogenous peptide antigen via MHC class II, regulation of defence response to virus by virus	0.000	1.019E-05	0.04471
OG0002461	protein kinase C epsilon type-like (LOC106589411)	protein serine/threonine kinase activity; ATP binding; protein phosphorylation; serine family amino acid metabolic process; intracellular signal transduction	cell adhesion, motility, and cell cycle, neuron growth, ion channel regulation, immune response, signal transduction	6.825E-08	6.478E-06	0.07476
OG0003986	solute carrier family 6 (neurotransmitter transporter), member 8 (slc6a8)	neurotransmitter:sodium symporter activity; cation transport; neurotransmitter transport; integral component of membrane	muscle contraction, required for the uptake of creatine in muscles and brain	7.970E-05	3.046E-12	0.05603
OG0000840	probable histone deacetylase 1-B (hda1b)	nucleus; NAD-dependent histone deacetylase activity (H3-K14 specific); metal ion binding;histone H3 deacetylation	transcriptional regulation, cell cycle progression, developmental events	0.0002432	1.952E-06	2.398E-12
OG0005675	E3 ubiquitin-protein ligase RNF128-like (LOC110537269), transcript variant X1	–	interleukin regulation, T-cell control, functions in the patterning of the dorsal ectoderm, sensitises ectoderm to respond to neural-inducing signals	0.0002432	1.282E-08	0.5357
OG0004474	endoplasmic reticulum aminopeptidase 1-like (LOC106570844)	endoplasmic reticulum membrane; proteolysis; regulation of blood pressure; zinc ion binding; antigen processing and presentation of endogenous peptide antigen via MHC class I; metalloaminopeptidase activity	regulation of innate immune response, response to bacterium, angiogenesis, regulation of blood pressure, fat cell differentiation	0.01917	2.008E-05	0.1651
OG0000651	tyrosine-protein phosphatase non-receptor type 6-like (LOC106576571), transcript variant X3	protein tyrosine phosphatase activity; protein dephosphorylation; tyrosine metabolic process	haematopoiesis, various immune functions	0.03186	1.547E-06	0.3844
OG0002139	calcipressin-2-like (LOC109900784), transcript variant X1	nucleic acid binding; calcium-mediated signaling	regulation of calcineurin-NFAT signalling cascade, calcium-mediated signalling, potential role in central nervous development	0.06629	2.753E-11	0.6235
OG0014882	junctophilin-2 (LOC111976619)	junctional membrane complex	calcium ion homeostasis, regulation of cardiac muscle tissue development	0.08725	3.460E-09	0.6595
Intensified & diversifying selection						
OG0000798	acetyl-CoA carboxylase beta (acacb), transcript variant X3	acetyl-CoA carboxylase activity; ATP binding; pyruvate metabolic process; fatty acid biosynthetic process; acetyl-CoA carboxylase complex; metal ion binding	regulation of cholesterol biosynthetic process, acetyl-CoA metabolic process	0.000	0.000	4.683
OG0011982	synapsin-3 (LOC111950806)	ATP binding; neurotransmitter secretion; synaptic vesicle	neurotransmitter secretion, regulation of synaptic transmission, GABAergic	0.0001606	0.000	8.330
OG0004141	clone Contig3164 cathepsin K precursor	proteolysis; cysteine-type peptidase activity	osteoclastic bone resorption, bone remodelling, collagen metabolic process	0.0002432	3.664E-14	7.769
OG0013215	lysine-specific demethylase 4B-like (LOC106574093), transcript variant X3	histone H3-K36 demethylation; histone H3-K9 demethylation (manually derived from UniProt/Swiss-Prot)	histone H3-K36 demethylation, histone H3-K9 demethylation, negative regulation of transcription	0.003614	3.970E-12	10.28
OG0003822	sterile alpha motif domain-containing protein 9-like (LOC109881303), transcript variant X2	protein binding	endosomal vesicle fusion	0.006278	9.533E-06	1.603
OG0007378	alpha-2,8-sialyltransferase 8F-like (LOC106588277), transcript variant X2	protein glycosylation; sialyltransferase activity	carbohydrate biosynthetic process, glycolipid metabolic process, ganglioside biosynthetic process, (modulation of cell signal transduction, maybe involved in function of nervous system)	0.008768	2.090E-11 9.549E-08	1.9625
OG0003820	paired amphipathic helix protein Sin3a-like (LOC106587652), transcript variant X3	transcription corepressor activity; transcription factor complex; negative regulation of transcription	negative regulation of transcription, regulation of lipid metabolic process, regulation of circadian rhythms	0.01812	5.292E-09	1.450
OG0004629	furin-1-like (LOC106587394), transcript variant X2	serine-type endopeptidase activity; proteolysis	involved in microbial infection, regulation of endopeptidase activity, TGF-beta-1 activation	0.01896	0.000	19.99
OG0010786	serine/threonine-protein kinase 6 (stk6)	protein serine/threonine kinase activity; ATP binding; protein phosphorylation; serine family amino acid metabolic process	activation of protein kinase activity, regulation of transcription	0.01972	0.000	6.183
OG0012826	beta-1,4-galactosyltransferase 6-like (LOC106560385), transcript variant X1	carbohydrate metabolic process; transferase activity	neuronal maturation, axonal and myelin formation, lactosylceramide biosynthetic process, carbohydrate metabolic process	0.03015	1.221E-12	6.768
OG0010420	torsin-1A-interacting protein 2 (LOC111975742)	endoplasmic reticulum organization; positive regulation of ATPase activity; protein localization to nuclear envelope (manually derived from UniProt/Swiss-Prot)	endoplasmic reticulum integrity, positive regulation of ATPase activity	0.03800	0.000	1.870
OG0011057	receptor-interacting serine/threonine-protein kinase 4-like (LOC106567956)	protein kinase activity; protein binding; ATP binding; protein phosphorylation	positive regulation of NF-kappaB transcription factor activity	0.08386	5.012E-06	3.356

Based on UniProt/Swiss-Prot gene information, the orthogroups with both signals of relaxed and diversifying selection are associated with functions such as immunity (5 of 9 orthogroups, e.g., ‘adaptor-related protein complex 1, mu 2 subunit (*ap1m2*)’), the nervous system (4 of 9 orthogroups, e.g., ‘protein kinase C epsilon type-like (*prkce*)’), muscle function (2 of 9 orthogroups, e.g., ‘solute carrier family 6 (neurotransmitter transporter), member 8 (*slc6a8*)’), blood pressure (1 of 9 orthogroups, ‘endoplasmic reticulum aminopeptidase 1-like (LOC106570844)’), and transcriptional regulation (1 of 9 orthogroups, ‘probable histone deacetylase 1-B (*hdac1-b*)’) (Table 2). This is in agreement with the more general GO term functions inferred using *Blast2GO* and associated tools (Fig. 6b – biological process GO terms, Table 2), such as neurotransmitter transport, calcium-mediated signalling, antigen presentation, regulation of blood pressure, and serine family amino acid metabolism.

Based on UniProt/Swiss-Prot gene information, orthogroups with both signals of intensified and diversifying selection are associated with transcriptional regulation (4 of 12 orthogroups, e.g., ‘paired amphipathic helix protein Sin3a-like (*sin3a*)’), lipid metabolism (3 of 12 orthogroups, e.g., ‘acetyl-CoA carboxylase beta (*acacb*)’), nervous system function (3 of 12 orthogroups, e.g., ‘synapsin-3 (*syn3*)’), carbohydrate metabolism (2 of 12 orthogroups, e.g., ‘alpha-2,8-sialyltransferase 8F-like (*st8sia6*)’), organelle function (2 of 12 orthogroups, e.g., ‘sterile alpha motif domain-containing protein 9-like (*samd9l*)’), bone growth (1 of 12 orthogroups, ‘cathepsin K precursor (*ctsk*)’), and immunity (1 of 12 orthogroups, ‘furin-1-like (*fur1*)’), amongst others (Table 2). Again, this agrees with the more general GO term functions inferred using *Blast2GO* and associated tools (Fig. 6c – biological process GO terms, Table 2), such as neurotransmitter secretion, negative regulation of transcription, serine family amino acid metabolism, fatty acid biosynthesis, and carbohydrate metabolism.

## Discussion

Our analyses of shared selection in the highly diversifying taxa *Coregonus* and *Salvelinus* identified genes and gene functions with deviating signatures of selection compared to five relatively less diversifying salmonid taxa and one non-salmonid species used as background. We identified more orthogroups under relaxed selection (*k* value < 1) than under intensified selection (*k* value > 1) (Figs. 2 and 6, Additional file 1). Further, we identified a set of 92 orthogroups that reflect signals of diversifying selection, nine and 12 of which additionally experienced either relaxed or intensified selection, respectively (Fig. 6, Table 2). We explored the underlying biological relevance and associated gene functions of these orthogroups under selection and observed trends of overrepresented GO terms in all three sets of orthogroups under selection (Fig. 4, Table 1). What is more, we identified 13 GO terms with *k* distributions deviating from the null expectation (Fig. 3).

In our functional analyses of the above selected orthogroups, genes in orthogroups under relaxed selection were found to be involved in processes such as transcriptional regulation, nervous system function, muscle and heart growth, lipid metabolism, and immunity, while orthogroups under intensified selection were found to be more often involved in signalling processes, actin filament-based processes, RNA splicing, carbohydrate metabolism, transcriptional regulation as well as nervous system and bone development (Additional file 1). Orthogroups under relaxed selection were enriched for ‘serine family amino acid metabolism’ while those under intensified selection tended to be enriched more strongly for various signalling pathways (Fig. 4, Table 1). ‘Regulation of transcription’ was overrepresented in orthogroups under both relaxed and intensified selection. Orthogroups with signals of diversifying selection showed a clear trend towards enrichment for signal transduction, transmembrane transport, proteolysis, lipid metabolism, and pyruvate metabolism GO terms (Fig. 4, Table 1) and often contained genes involved in transcriptional regulation, development, lipid metabolism, and immunity (orthogroups under diversifying selection, Additional file 2, including associated GO terms). Therefore, our results imply different intensities of selection on different gene functions and processes.

### Selection parameter distribution – shift to relaxation?

Our results agree with the repeated finding of a relaxation of selective constraint in rapidly diverging taxa [18, 19, 72, 73]. One possible cause behind this observation could be an increase of ecological opportunity due to the emergence of new habitat and a decrease in interspecies competition, for example in caves [72] or within postglacial lakes [74–79]. The adaptation to life in lakes, before or in the course of (repeated) postglacial colonisations, such as in charr and whitefish, could be such an ecological opportunity, entailing a release of selective pressures on a substantial number of genes.

From the above results we can also conclude that relaxation of selective constraints does not affect the whole set of protein-coding genes to the same degree. Purifying selection and selective constraint still affect a large number of genes with functions that ensure an organism’s integrity, survival, and reproduction (see orthogroups under intensified selection) [80]. However, in charr and whitefish we observed a larger number of orthogroups under relaxed selection that therefore escaped the effects of purifying selection.

The observation of largely different GO terms in orthogroups under relaxed selection or intensified selection agrees with a large number of studies that demonstrated different selective pressures on different gene functions [81, 82]. Various functional and structural constraints govern the substitution patterns within genes. For example, amino acid alterations in some groups of proteins can easily make them dysfunctional [81], while other kinds of proteins are under less constraint to vary [83] or are even beneficial to fitness when being different from the majority of the population (i.e., negative frequency-dependent or balanced selection) [84–86]. The observation of intensified selection on DNA repair in our dataset and the overrepresented GO terms related to oxidative stress and apoptotic signalling, for example, could highlight the importance of an effective response to environmental stress in rapidly diversifying taxa. Also, carbohydrate and ribosome-related processes seem to be under strong selective constraint. On the other hand, relaxation of selective constraint on ATPase activity and proton transmembrane transport as well as various lipid metabolism genes, amongst others, could have led to the physiological variety observed in extant highly diversifying salmonids.

### Gene functions under relaxed selection in *Coregonus* and *Salvelinus*

Many of the orthogroups under parallel relaxed selection, i.e. those that experienced a release of selective constraint in *Coregonus* and *Salvelinus*, have been linked to the divergence of these species along the benthic-limnetic axis within lakes found in earlier research [74, 87–91]. Examples include the ‘peripherin-2-like’ gene, which has been implicated in visual adaptation in *S. namaycush* in Lake Superior [92] and the ‘natterin-3-like’ gene, which plays a putative role as an immunopeptide and experienced pronounced expression divergence in Icelandic *S. alpinus* ecomorphs [93, 94]. Based on our analyses, we propose these are linked to the evolution of novel phenotypes and the variation in feeding and swimming behaviour, energy storage, and the release of competition in postglacial lakes (behaviour, muscle and heart growth, locomotion, lipid metabolism, nervous system function) as well as a release or change of parasite and pathogen burden (immunity genes) in postglacial lakes [95]. This implies changes in selective pressure on these gene functions as a consequence of shifts in the ecology of highly diversifying salmonids.

### Gene functions under intensified selection in *Coregonus* and *Salvelinus*

Genes under intensified selection are expected to have a strong role for survival and reproduction (i.e., fitness) of organisms [49]. We found the orthogroups under intensified selection showed a high number of processes associated with key metabolic pathways (mainly of carbohydrates and lipids), signal transduction, and regulation of gene expression and the immune system, indicating strong selective pressures on these processes and functions in *Coregonus* and *Salvelinus*. The occurrence of immunity genes under intensified selection in our results would rather imply changing parasite and pathogen regimes rather than a complete release of pathogen burden [96]. Interestingly, our results also indicated strong selective pressures on actin filament-based processes and RNA splicing as well as protein modification, which are functions with a crucial role in development [96–99]. Among these genes, some were found to be directly linked to differences in bone development [e.g., ‘paired like homeodomain 1’ and ‘cathepsin K precursor’) [100–102]. We speculate that these developmental process genes may be relevant in the extreme morphological diversity that can be found among closely related *Coregonus* species and particularly *Salvelinus* species [90, 100–104].

### Gene functions under diversifying selection in *Coregonus* and *Salvelinus*

Diversifying (i.e., positive) selection on genes, which in this context means that selective pressure favours amino acid polymorphism in a particular gene, affected 92 orthogroups in *Coregonus* and *Salvelinus* relative to the non-diversifying taxa. The enrichment of lipid, glycerolipid, and pyruvate metabolism genes is a strong indication that these metabolic processes experienced diversification/positive selection in *Coregonus* and *Salvelinus*, which could imply a role in ecological diversification and habitat shifts across the depth axis in lakes. Lipid metabolism has been commonly found to be under diversifying selection in freshwater fishes, such as sticklebacks [105] and salmonids [89, 106]. Also, diversifying/positive selection on transmembrane transport, for example, has been shown repeatedly in teleost fish adapting to the freshwater environment, such as sculpin and alewife, putatively because of its role in osmoregulation [107, 108].

### Overlap between selection types

A combination of approaches, such as *aBSREL* and *RELAX* as we apply here, has been shown to reliably infer genes under selection and is also able to appropriately distinguish between positive selection and relaxed selection, which are hard to disentangle using single conventional methods [109–111]. This is particularly important since it has been known for some time that the number of false positives in tests for positive or diversifying selection can be substantial [112–115]. Overlapping sets of orthogroups under selection inferred with different analytical approaches, as we resolve here, can therefore implicate consistent, strong, and specific signals of selection. For example, orthogroups with signals of relaxed and diversifying selection likely experienced a release of purifying selection, with the potential for evolutionary diversification [19, 49, 116].

One of the orthogroups with the most extreme signal of relaxed and diversifying selection was ‘adaptor-related protein complex 1, mu 2 subunit (*ap1m2*)’ (Table 2). Apart from a role in endothelial and intestinal immunity [117], *ap1m2* plays a central role in the development of endoderm-derived organs (e.g., stomach and intestine) in zebrafish [118]. Given the differing parasite pressure [119, 120] and the diversity of trophic niches in lake systems [121, 122], genes under reduced selective constraint could play a crucial role in phenotypic diversity in the often lake-dwelling *Salvelinus* and *Coregonus*. Another gene showing a strong signal of relaxed and diversifying selection was ‘solute carrier family 6 (neurotransmitter transporter), member 8 (*slc6a8*)’, which regulates creatine uptake in tissues with high energy demands such as muscle and brain tissue [122–124]. Recent research on *C. mykiss* and *C. maraena* found two copies of this gene, which were either mainly expressed in muscle or kidney tissue, with the strongest expression overall in muscle [125]. Changes of selection regimes on these functions are expected, given that *Salvelinus* and *Coregonus* experienced a diversification in trophic and swimming behaviour, ecology, and life history (e.g., *Salvelinus* and *Coregonus* can be free swimming, lake bottom dwelling, or restricted to the littoral zone of lakes). Our analyses independently corroborate an important functional role of this gene in highly diversifying salmonids.

Orthogroups showing both intensified and diversifying selection likely constitute targets of “true” positive selection rather than relaxed selection misinterpreted as positive selection. Genes in these orthogroups are thus under stronger selective pressure and are potentially of higher importance to survival and overall fitness [49] in our analyses of *Coregonus* and *Salvelinus*. Two of the most strongly significant orthogroups under intensified and diversifying selection involved the genes ‘synapsin-3 (*syn3*)’ and ‘cathepsin K precursor (*ctsk*)’. *syn3* is involved in neurotransmitter secretion [126] and plays a crucial role in early neuronal differentiation and in neuronal progenitor cell development in fish [127] as well as in mammals [128]. *ctsk*, encoding a collagenase, has an important function in bone modelling and remodelling as well as bone homeostasis in vertebrates [129, 130] and is differentially expressed in *S. alpinus* [102]. Both *syn3* and *ctsk* are among the genes experiencing the highest selective pressure in *Coregonus* and *Salvelinus* as compared to other salmonid taxa, which is an indication of their importance for survival in these two genera and warrants further research.

Not only specific genes but also the gene functions associated with orthogroups under selection were found to have biological relevance. Changes in heart and muscle function, immunity genes, lipid metabolism, and transcriptional regulation have previously been implicated in diversification in salmonids [81, 131–136], cichlids [33], ants [83], and tonguefishes [84]. Therefore, our findings from molecular evolution agree with evidence from ecomorphological divergence of *Coregonus* and *Salvelinus* of immune response, feeding, metabolism, and locomotion [46, 137, 138].

### Whole-genome duplication and potential effects on relaxed selection

Multiple lines of evidence suggest that whole-genome duplication (WGD) events give rise to vast amounts of genetic material that can subsequently experience elevated substitution rates and relaxation of selective constraints [139–141]. The lineage ancestral to salmonids experienced a WGD around 80–103 Mya [67–69]. It has been speculated that this may have contributed to new phenotypic innovations and to the ecological success of salmonids [67, 142–145], although a contribution to diversification rates is contentious [67, 146]. While conventional approaches for the detection of positive and relaxed selection based on orthologues in salmonids would potentially be biased in the case of duplicated and non-rediploidised genes, approaches based on orthogroups, as we applied here, alleviate this issue by explicitly comparing selection shared in *Coregonus* and *Salvelinus* relative to all other salmonid lineages [58]. This approach circumvents the difficulty of inferring orthologues in taxa that experienced multiple duplications and unequal rediploidisation.

While genes duplicated during the salmonid WGD might be more susceptible to relaxed selection [49, 147], charr and whitefish are in different subfamilies and not sister genera (Fig. 1) [41, 148]. Therefore, based on evolutionary relationships, they should have rediploidisation patterns more similar to their sister genera than to each other and there is no reason to suspect unequal rediploidisation among genera to be the cause of the relaxed selection signals we identified in *Coregonus* and *Salvelinus*. It is more likely that these two genera experienced similar selective pressures due to common diversification patterns, environmental conditions, or other common ecological or life history traits. Our results from molecular evolution analyses reflect these shared ecological and evolutionary patterns. However, recent results on gene expression in duplicated genes of salmonids have shown that relaxed selection often occurs in downregulated gene duplicates [149], which might be associated with dosage balance effects [150, 151]. Therefore, future research should ideally analyse selection in concert with gene expression and duplication.

## Conclusions

Our findings suggest that there is some evidence for a parallel relaxation of selective constraint in the repeatedly diversifying salmonid lineages *Coregonus* and *Salvelinus* compared to all other salmonid lineages, which are known to be less diversifying. Genes in orthogroups under relaxed selection are involved in functions with a potential role in the rapid diversification and ecological adaptation that can be observed in wild populations of *Coregonus* and *Salvelinus* and experienced a release of selective pressure, including genes involved in behaviour, muscle function, and infection. On the other hand, orthogroups under intensified selection, such as various signal transduction and regulatory genes, are under stronger selective pressure and are consequently expected to have higher fitness effects in charr and whitefish compared to other salmonids. An independent analysis of orthogroups under diversifying selection showed an enrichment for signal transduction genes and GO terms of various metabolic processes, while genes involved in DNA binding were underrepresented. Orthogroups with both signals of relaxed and diversifying or intensified and diversifying selection can give further hints as to what selective processes govern the evolution of these genes and are also important candidates for gene sets under particularly weak (relaxed) selective pressure (while still experiencing molecular diversification), or strong (intensified) selective pressure. The discovered orthogroups under selection are a valuable resource for further investigations into the importance of certain genes for rapid diversification in salmonids and beyond.

## Material & methods

### Taxonomic dataset

All analyses were performed on RNAseq transcriptome datasets drawn from a search in NCBI and a literature search in Google Scholar (keywords: each salmonid genus and species, in combination with “transcriptome” and/or “RNAseq” or “RNA-seq”) to identify all available studies. The final dataset represents one transcriptome per species of transcriptomes of all available taxa as of October 2016, with additional in-house data for *Salmo salar*, *Salmo trutta*, *Salvelinus alpinus*, and *Coregonus lavaretus* that was published at a later point [88]. We aimed to retain transcriptomes with a maximal number of overlapping transcripts across salmonids, which consisted of nine species representing all five major genera (*Coregonu*s, *Oncorhynchus*, *Salmo*, *Salvelinus*, and *Thymallus*), and the closest relative as outgroup (*Esox lucius*) (Fig. 1, Table 3). The selected transcriptome assemblies were all derived from several tissues so as to obtain almost complete sets of transcripts (see references in Table 3). The topology for foreground branch definitions is based on the phylogenetic tree shown in Fig. 1, which was obtained using maximum-likelihood tree estimation based on a preliminary set of 1564 orthologues (derived from the same set of transcriptomes; orthologue alignments available upon request) and in agreement with the current understanding of salmonid phylogenetic relationships [41, 148]. From the full dataset, ranging from 59.0 to 209.2 Mbp per transcriptome (Table 3), we obtained 2702 sequences of orthogroups (mean number of orthologues per orthogroup: 4.77; mean length 1783 bp; range 607–14,743 bp; total 4.82 Mbp). Using a *Blast2GO* [156] analysis (for details see below), we confirmed that the dataset was not enriched for any particular GO categories (Fisher’s Exact Test: all *p* > 0.10) and therefore not a biased representation of transcripts.

**Table 3:** Sources of transcriptome assemblies used in this study

Transcriptomes				
*Species*	*Source/Accession no.*	*Size [Mbp]*	*Transcript count*	*Reference*
*Coregonus clupeaformis* (lake whitefish)	PhyloFish (phylofish.sigenae.org, last access: 05.10.2016)	158.5	66,996	[152]
*Esox lucius* (northern pike)	GATF00000000.1 (NCBI GenBank)	188.7	55,424	[153]
*Oncorhynchus mykiss* (rainbow trout)	Salmon Transcriptome website (salmon.cgrb.oregonstate.edu, last access: 05.10.2016)	209.2	130,599	[154]
*Oncorhynchus tshawytscha* (Chinook salmon)	GSE59756 (NCBI Gene Expression Omnibus)	159.8	183,740	[155]
*Salvelinus fontinalis* (brook trout)	PhyloFish (phylofish.sigenae.org, last access: 05.10.2016)	166.0	69,441	[152]
*Thymallus thymallus* (European grayling)	PhyloFish (phylofish.sigenae.org, last access: 05.10.2016)	160.9	67,157	[152]
*Coregonus lavaretus* (European whitefish)	earlier version of GGAO00000000.1 (NCBI GenBank; made accessible upon request)	59.0	44,348	[88]
*Salvelinus alpinus* (Arctic charr)	earlier version of GGAP00000000.1 (NCBI GenBank; made accessible upon request)	59.5	44,354	[88]
*Salmo salar* (Atlantic salmon)	earlier version of GGAQ00000000.1 (NCBI GenBank; made accessible upon request)	63.0	44,554	[88]
*Salmo trutta* (brown trout)	earlier version of GGAR00000000.1 (NCBI GenBank; made accessible upon request)	62.0	46,308	[88]
Genome				
*Species*	*Source/Accession no.*	*Size [Mbp]*	*Gene count*	*Reference*
*Salmo salar* (Atlantic salmon)	GCF_000233375.1 (NCBI RefSeq assembly)	77.5	48,589	[68]

### Orthogroup and OG tree inference

The longest open reading frames (ORFs) in the transcriptome sequences of the ten species (excluding mitochondrial genes due to different genetic codes that could bias the selection analyses) and, additionally, protein-coding genes of the salmon genome (NCBI RefSeq assembly accession: GCF_000233375.1) [68] were determined using *TransDecoder* v2.1 (https://github.com/TransDecoder/TransDecoder). Next, the standard mode of *OrthoFinder* v2.2.3 [157], which uses a five-step algorithm that mitigates gene length bias and other biases introduced by other methods, was used to infer orthogroups from the *TransDecoder* ORFs. Based on the *OrthoFinder* orthogroup assignments, gene sequences were then extracted from the whole transcriptomes with custom *Python* scripts (*Python v3.6.5*) if they were also present in the protein-coding sequences of the Atlantic salmon genome v2 (NCBI RefSeq assembly accession: GCF_000233375.1) [68] to exclude spurious transcripts. To further minimise the number of spurious transcripts retained, only orthogroups with a higher or equal number (but not larger than four) of orthologous genes in the focal species compared to the salmon genome were included to avoid inclusion of collapsed orthologues (i.e., multiple genes that were merged into one during assembly, which seems to be a common issue in the salmonid transcriptomes published so far according to in-house results and A. Yurchenko and M. Carruthers, personal communication). Orthologues were then combined into orthogroups using custom *Python* scripts if they matched the same gene sets in the salmon reference genome. Duplicated orthogroups, inferred as multiple occurrences of the same orthologue sets, orthogroups with a number of sequences exceeding 80, which could include an inflated number of duplicates, or lower than seven, to avoid orthogroups unrepresentative of the whole dataset, and those with fewer than seven taxa were removed with custom *Python* scripts. The longest ORFs with a minimum length of 200 bp were then extracted from this orthogroup set with get_orfs_or_cdss.py (https://github.com/peterjc/pico_galaxy/blob/master/tools/get_orfs_or_cdss/get_orfs_or_cdss.py). A codon-based alignment was then produced using *PRANK* v.140603 [158]. The aligned orthogroups were then trimmed and filtered with *trimAl* (−resoverlap 1.0, −seqoverlap 0.38 -noallgaps) [159], optimising both alignment quality and the number of retained alignments. Using the final set of orthogroups as input for *FastME* [160], we then constructed phylogenies of each orthogroup separately for use in the selection analyses below. The number of retained genes per orthogroup was calculated using custom *BASH* (*BASH v3.2*) and *Python* scripts.

### Selection analyses

Foreground branches for subsequent selection analyses were labelled using a custom *R* script (*R v3.5.1*) and the *R* package *ape* [161–163]. All selection analyses were then performed in *HYPHY v2.3.13.20180521beta(MP)* [48]. Orthogroup alignment FASTA file headers were shortened using custom *Python* scripts and FASTA and tree files were combined with custom *BASH* scripts. Using the above foreground definitions, *RELAX* [49], included in the *HYPHY* package, was run to infer orthogroups with significant signals of relaxed or intensified selection in the foreground branches relative to the background branches and to infer the selection strength parameter *k* (as defined in [49]). The software *aBSREL* (adaptive Branch-Site Random-Effects Likelihood inference) [50], also contained in the *HYPHY* package, was then used to obtain orthogroups with a proportion of sites under significant diversifying or positive selection in the foreground branches relative to the background branches. Relevant information from both the *RELAX* and *aBSREL* output was extracted with custom *BASH* scripts. Multiple testing was accounted for with false-discovery rate (FDR) correction [164] or Bonferroni correction [165, 166] in *R*. We report on the FDR level of significance throughout the manuscript but also report the orthogroups under selection after Bonferroni correction in Additional file 1. Significant differences in the number of orthogroups under relaxed or intensified selection were quantified using Fisher’s Exact Tests in *R*. A two-sided one-sample Wilcoxon signed-rank test in *R* was used to infer whether the overall selection parameter (*k*) distribution differed significantly from the null expectation of 1. Selection parameter distributions were visualised using the *R* package *ggplot2* [167]. Hypergeometric expectations of orthogroups under multiple types of selection were calculated in *R*.

### Gene ontology analyses

GO terms were inferred using a complementary set of methods in *Blast2GO v5.2.5* [156]. We used *BLASTn* (*megablas*t) [168, 169] using *QBlast* (NCBI Blast Server) and standard web blast against non-redundant nucleotide sequences and in a second step against protein sequences. A BLAST expectation value (*e*-value) threshold of 1.0E-3 was used. Only the top blast hit was retained per orthogroup BLAST search. BLAST descriptors were extracted for later annotations. *BLASTn* was run with a word size of 28, a low complexity filter, a high-scoring segment pair (HSP) length cutoff of 33, and an HSP-hit coverage of 0. GO mapping was performed with the *Goa version 2018.12* database [170]. Orthogroups were remapped with *InterProScan* [171–173] using *EMBL-EBI InterPro* to improve mapping success. Annotations were then created using an annotation cut-off of 55, GO weight of 5, an *e*-value filter of 1.0E-6, an HSP-hit coverage cut-off of 0, and a hit filter of 500. Further annotations were obtained using merging of *InterProScan* GOs with existing annotations. Annotations were then augmented by *ANNEX* [174]. Remaining unannotated sequences were annotated using blast descriptions, applying a minimum similarity of 85 and validation of annotations. Gene functions were inferred based on *Blast2GO* and Uniprot/Swiss-Prot gene information for fish, mice, and humans (last accessed on 27/01/2019).

GO term enrichment analyses were performed using Fisher’s Exact Tests in *Blast2GO* on the whole dataset and all sets of orthogroups under selection (from *RELAX* and *aBSREL* with Bonferroni multiple correction, FDR correction, or no correction) with FDR multiple correction (FDR threshold of 0.10). GO terms were extracted from the *Blast2GO* output using custom *R* scripts. Overlap of sets of orthogroups under selection was determined with custom *R* scripts. Additionally, GO term enrichment was tested using custom *R* scripts to see trends in over- and underrepresentation (*Blast2GO* only outputs significantly over- and underrepresented GO terms after FDR correction). Violin plots of selection parameter *k* distributions, compared across GO terms, were plotted with the *ggplot2 R* package. The significance of deviations from the null expectation of *k* = 1 was tested for each GO term using two-sided one-sample Wilcoxon signed-rank tests in *R*.

To summarise and visualise the obtained GO terms in the different sets of orthogroups under selection, we used the *REVIGO* online analysis tool with an allowed similarity score of GO terms of 0.9, the zebrafish reference GO term database available in *REVIGO*, and SimRel as semantic similarity measure (last access: 01.10.2019) [70], which makes use of a semantic clustering algorithm and the *p*-values from the GO term enrichment analysis in *R* above. In this context, semantic similarity refers to the degree of relatedness between two entities/GO terms by the similarity in meaning of their annotations [175]. *REVIGO* clusters GO terms of sets of genes based on this algorithm in a two-dimensional semantic similarity space. Additionally, *REVIGO* ranks the GO terms of genes according to their redundancy.

### Gene set enrichment analysis

Gene set enrichment analysis (GSEA) was performed on gene symbols of zebrafish homologues of human genes derived from *Blast2GO*. Zebrafish homologues were obtained from http://www.informatics.jax.org/downloads/reports/HOM_AllOrganism.rpt using a custom *R* script. The selection parameter (*k*) values from the above *RELAX* analysis were transformed into -1 to 1 scores in *R*, with negative values representing relaxed selection orthogroups and positive values representing intensified selection orthogroups. We then used *WebGestalt* [176–179] on the zebrafish gene symbols and associated transformed *k* scores to derive a) the top ten enriched biological process GO terms and b) the top ten enriched KEGG pathways in both the relaxed and intensified orthogroups. We performed 10,000 permutations for each GSEA run. All other parameters were kept at default settings (last use: 02.10.2019).

## Supplementary information

**Additional file 1.** Total set of orthogroups with associated GO terms and results of relaxed and intensified selection analyses.

**Additional file 2.** Orthogroups under diversifying selection (FDR < 0.10) in both *Coregonus* and *Salvelinus*.

## Data Availability

The scripts used for orthogroup inference, selection, and gene ontology analyses, the scripts for intermediate steps and running software, and the orthogroup alignments are made publically available in the University of Glasgow Enlighten repository, DOI: 10.5525/gla.researchdata.913. The transcriptomes and genomes used are from the National Center for Biotechnology Information (NCBI; https://www.ncbi.nlm.nih.gov/) (GenBank accession: GATF00000000.1, Gene Expression Omnibus accession: GSE59756, RefSeq assembly accession: GCF_000233375.1), the Oregon State University’s Salmon Transcriptome website (salmon.cgrb.oregonstate.edu; *Oncorhynchus mykiss*), the PhyloFish project website (phylofish.sigenae.org; *Coregonus clupeaformis*, *Salvelinus fontinalis*, *Thymallus thymallus*, last access: 05.10.2016), and in-house data (earlier versions of NCBI GenBank accessions: GGAO00000000.1, GGAP00000000.1, GGAQ00000000.1, GGAR00000000.1; available upon request).

## Abbreviations

aBSREL: Adaptive branch-site random effects likelihood
AGE: Advanced glycation end products
BASH: Bourne-again shell
BLAST: Basic local alignment search tool
BLASTn: Nucleotide basic local alignment Search Tool
bp: Base pair
dN/dS: Ratio of the number of nonsynonymous substitutions per nonsynonymous site to the number of synonymous substitutions per synonymous site
DNA: Deoxyribonucleic acid
*e*-value: Expectation value in BLAST search
FASTA: Text-based format for representing nucleotide and amino acid sequences
FDR: False discovery rate
FOX: Forkhead box
GO: Gene ontology
GSEA: Gene set enrichment analysis
HSP: High-scoring segment pair
KEGG: Kyoto Encyclopedia of Genes and Genomes
Mbp: Megabase pair
Mya: Million years ago
NCBI: National Center for Biotechnology Information
ORF: Open reading frame
RAGE: Receptor for advanced glycation end products
RELAX: Hypothesis testing framework for detection of relaxed selection
RNAseq: Ribonucleic acid sequencing
Swiss-Prot: An annotated protein sequence database
UniProt: Universal protein resource
WGD: Whole-genome duplication
ω: Synonymous to dN/dS
